# Association between soluble α-klotho and mortality risk in middle-aged and older adults

**DOI:** 10.3389/fendo.2023.1246590

**Published:** 2023-08-24

**Authors:** Min-Hsiang Chuang, Hung-Wei Wang, Yun-Ting Huang, Ming-Yan Jiang

**Affiliations:** ^1^ Renal Division, Department of Internal Medicine, Chi Mei Medical Center, Tainan, Taiwan; ^2^ Renal Division, Department of Internal Medicine, Chi Mei Hospital Chiali, Tainan, Taiwan; ^3^ Department of Pharmacy, Chia Nan University of Pharmacy & Science, Tainan, Taiwan

**Keywords:** klotho, aging, all-cause mortality, chronic kidney disease (CKD), hepatitis C virus (HCV)

## Abstract

**Introduction:**

Studies on association of α-klotho levels with mortality risk in general population are relatively scarce and inconclusive. Therefore, we conducted a population-based cohort study to investigate the relationship between soluble α-klotho and all-cause mortality in a nationally representative sample of middle-aged and older adults in the United States (U.S.).

**Methods:**

The study population was 2007-2016 National Health and Nutrition Examination Survey (NHANES) participants, totaling 13,583 adults aged 40-79 years. Participants were divided into 7 groups by septile of α-klotho levels. We linked the NHANES data to the National Death Index to determine participants’ survival status. End of follow-up was participants’ death date or December 31, 2019.

**Results:**

We observed that males, current smokers, older age, higher body mass index, and lower estimated glomerular filtration rate correlated to lower α-klotho levels, while hepatitis C virus infection correlated to higher α-klotho. The population mortality rate was 11.8 per 10,000 person-months (1,490 deaths); group 1 (the first septile) had higher mortality risk compared with group 2 through group 7. By weighted Cox regression with adjustment for potential confounders, we found that group 2 through group 6, but not group 7, were associated with 25% to 35% lower risk of all-cause mortality compared with group 1. When compared with group 4, we observed that both group 1 (HR: 1.46, 95% CI 1.13-1.88) and group 7 (HR: 1.38, 95% CI 1.09-1.74) were associated with higher mortality risk.

**Conclusion:**

In summary, among middle-aged and older U.S. adults, we observed a non-linear association between soluble α-klotho and all-cause mortality, with individuals at the two extremes at increased risk of death.

## Introduction

Alpha-klotho is an anti-aging protein involved in the regulation of oxidative stress, inflammation, cellular senescence, and multiple signaling pathways (1, 2). Preclinical studies on animal models had demonstrated that α-klotho possesses antioxidative and anti-inflammatory properties by decreasing reactive oxygen species (ROS) activity and suppressing interleukin (IL-) 6, IL-1β, and tumor necrosis factor alpha (TNF-α) levels, and was potentially protective against diseases such as contrast-induced acute kidney injury, pulmonary epithelial damage from hyperoxia, valvular fibrosis, and cardiac dysfunction in cardiorenal syndrome (3–6). In the field of cardiovascular disease, α-klotho was postulated to play roles in the regulation of myocardial remodeling and aging of the vasculature (7, 8). Such antioxidative and protective effect of α-klotho against cell injury was also supported by *in vitro* and *in vivo* human studies (9, 10). Among patients undergoing non-emergent coronary angiography, reduced soluble α-klotho level was found to correlate with the presence and severity of coronary artery diseases (11). Similarly, in patients with chronic kidney disease (CKD), an inverse correlation between soluble α-klotho level and left ventricular mass index had been observed (12). In cancer research, soluble α-klotho was proposed to act as a tumor suppressor by regulating cell metabolism and modulating multiple oncogenic pathways (13, 14). Therefore, research on the potential therapeutic roles of klotho in oncology, renal and cardiovascular diseases is emerging, and some beneficial effects of treating klotho deficiency have been observed in animal models (14, 15).

Several epidemiological studies had shown that in older community-dwelling adults, low serum α-klotho concentrations were associated with poor health outcomes including poor skeletal muscle strength (16), disability in activities of daily living (17), and increased risk of all-cause mortality (18). In addition, in older adults with CKD, higher serum α-klotho levels have been associated with slower decline in kidney function (19). Furthermore, among individuals with advanced kidney disease or those receiving renal replacement therapy, evidence suggested that lower α-klotho levels were linked to an increased risk of all-cause mortality (20).

Although previous studies have shown the associations between α-klotho levels and various health outcomes, evidence on the association of α-klotho with the risk of death in the general population is relatively scarce. A recent study using nationally representative data from the National Health and Nutrition Examination Survey (NHANES) in the United States (U.S.) reported a null association between serum α-klotho level and all-cause mortality in adults (21). However, individuals with marked impairment of kidney function were also included in that study. While poor kidney function significantly correlated with low levels of soluble α-klotho (22), the inclusion of these individuals in the analysis may have biased the study results. Additionally, the follow-up time in that study was relatively short with a mean of 57.7 months, which might fail to capture the long-term results. Accordingly, our study aims to investigate the association between soluble α-klotho level and long-term mortality risk among adults with estimated glomerular filtration rate (eGFR) ≥ 30 mL/min/1.73 m^2^ using the updated data from the NHANES.

## Materials and methods

### Data source

NHANES consists of a series of cross-sectional, multistage probability sampling for the population of U.S. civilian not residing in institutions, which is conducted in 2-year cycles since 1999 by the National Center for Health Statistics (NCHS) (https://wwwn.cdc.gov/nchs/nhanes/tutorials/module2.aspx). NHANES data are gathered from survey participants through questionnaires on health-related topics, physical examination, and laboratory tests, and are available to the public on the NCHS website (https://www.cdc.gov/nchs/nhanes/index.htm). All NHANES participants provided written informed consent. The NHANES protocols were approved by the research ethics review board of the NCHS.

### Study population

Soluble α-klotho testing was eligible for serum samples from 40- to 79-year-old participants in NHANES 2007-2008, 2009-2010, 2011-2012, 2013-2014, and 2015-2016 cycles. Therefore, for this population-based cohort study, we merged 5 discrete 2-year cycles of the continuous NHANES (2007-2008 through 2015-2016 cycles), which resulted in a total of 17,389 participants aged 40 to 79 years. We excluded individuals without soluble α-klotho testing (n=3,625), whose survival status was not available (n=16), who had received dialysis in the past 12 months (n=57), who had no serum creatinine data (n=5), or who had eGFR < 30 mL/min/1.73 m^2^ (n=103). Finally, the study cohort consisted of 13,583 adult participants aged 40 to 79 years.

### Exposure variables

The exposure variable of interest was the level of soluble α-klotho, which was measured using a commercially available ELISA kit manufactured by IBL International in Japan. We divided the participants into 7 groups based on the septile of soluble α-klotho levels: group 1 (n=1,940) with α-klotho levels of ≤ 575.8 pg/ml, group 2 (n=1,943) of 575.9 to 678.8 pg/ml, group 3 (n=1,939) of 678.9 to 761.0 pg/ml, group 4 (n=1,944) of 761.1 to 849.9 pg/ml, group 5 (n=1,939) of 850.0 to 961.3 pg/ml, group 6 (n=1,942) of 961.4 to 1140.0 pg/ml, and group 7 (n=1,936) of ≥ 1140.1 pg/ml. As there is no established reference range for α-klotho levels, we chose septiles to explore the potential non-linear association without complicating the presentation of results.

### Outcome variables

The outcome of interest was all-cause mortality. We linked NHANES data to death records from the National Death Index to obtain the participants’ survival status. The International Classification of Diseases-Tenth Revision was used to define the cause of death, which were determined from the primary causes of death contained in publicly available NHANES linked mortality files. The follow-up period for each participant started from the date of the NHANES baseline interview until the date of the participant’s death or last follow-up (December 31, 2019).

### Covariates

Race/ethnicity was classified as non-Hispanic White people, non-Hispanic Black people, Hispanics, and other races (including multi-racial) based on self-identification. The family income-to-poverty ratio was determined by dividing total family income by the corresponding poverty threshold for the specific family size, appropriate year, and state. This ratio was classified into 3 categories as <1.3 (low income), ≥1.3 to < 3.5 (middle income), and ≥3.5 (high income). Body mass index (BMI) was computed by dividing the body weight in kilograms by the square of height in meters and was classified into 3 categories as < 25 (normal), ≥ 25 to < 30 (overweight), and ≥ 30 kg/m^2^ (obese). The eGFR was calculated using the 2021 Chronic Kidney Disease Epidemiology Collaboration (CKD-EPI 2021) creatinine equation. Diabetes and hypertension were identified through self-reported diagnosis with the disease or usage of medications. Cardiovascular disease (CVD) was defined by self-reported history of congestive heart failure, coronary heart disease, angina, or heart attack. Previous stroke was defined by self-reported history of the disease. History of hepatitis C virus (HCV) infection was defined by HCV seropositivity or detectable HCV RNA.

### Statistical analysis

The characteristics of the sampled population were described using survey-weighted means and standard errors (SE) or counts and survey-weighted proportions. We conducted survey-weighted linear regression analyses to examine the association of covariates with soluble α-klotho levels. We used Kaplan-Meier method with Log-Rank test to plot the survival curve by septile of serum α-klotho levels. Survey-weighted Cox regression analysis was conducted to investigate the association between levels of soluble α-klotho and the mortality risk with adjustment for potential confounding variables. In model 1, we adjusted for age and sex. In model 2, we added self-identified race/ethnicity, BMI, survey cycle, eGFR, diabetes and hypertension in addition to the variables in model 1. In model 3, we further added CVD, previous stroke, history of HCV infection, and smoking status in addition to the variables in model 2. Finally, in the model 4, we added family income-to-poverty ratio to the regression model. Data were presented in the form of hazard ratio (HR) and 95% confidence interval (CI). To evaluate the proportional hazards assumption, time-dependent covariates was included in the Cox model and showed no violation of the assumption. We also performed survey-weighted multinomial logistic regression analysis to compare the characteristic differences in serum α-klotho status across groups. Data were presented as odds ratio (OR) and 95% confidence interval (CI). Statistical computation was performed using SAS 9.4.

## Results

The population aged 56.1 ± 0.2 years (survey weighted mean ± standard error) and 47.8% of them were male. The race/ethnicity distribution included 73.1% White people, 9.1% Black people, 11.3% Hispanics, and 6.5% other races/ethnicities (**Table 1**). In addition, three quarters of the population were overweight or obese, and nearly half were never smokers (**Table 1**). Using weighted multiple linear regression, we found that females, non-Hispanic Black people (vs. non-Hispanic White people), individuals with higher eGFR, never or former smokers, and those with a history of HCV infection positively correlated to higher α-klotho levels. In contrast, individuals with higher BMI and older age were associated with lower α-klotho levels. Diabetes, hypertension, CVD, and previous stroke were not significantly associated with soluble α-klotho levels (**Table 2**).

**Table 1:** Baseline characteristics of participants by septile of soluble α-klotho levels.

	Total	S1	S2	S3	S4	S5	S6	S7
No. of participants	13583	1940	1943	1939	1944	1939	1942	1936
** Age** (years old)	56.1 ± 0.2	57.7 ± 0.3	56.9 ± 0.3	56.2 ± 0.3	56.0 ± 0.3	55.9 ± 0.3	55.5 ± 0.4	54.5 ± 0.3
40-49 years old	3804 (31.7%)	459 (27.0%)	507 (31.3%)	534 (31.6%)	553 (31.7%)	559 (32.3%)	574 (32.6%)	618 (35.8%)
50-59 years old	3616 (31.3%)	492 (29.3%)	457 (28.3%)	534 (30.4%)	531 (33.3%)	506 (31.0%)	541 (32.8%)	555 (34.5%)
60-69 years old	3790 (23.8%)	572 (27.3%)	556 (23.4%)	543 (26.1%)	516 (21.8%)	552 (23.8%)	544 (24.1%)	507 (19.7%)
70-79 years old	2373 (13.2%)	417 (16.4%)	423 (17.0%)	328 (11.9%)	344 (13.3%)	322 (12.9%)	283 (10.5%)	256 (10.0%)
Sex								
Male	6583 (47.8%)	989 (48.2%)	1011 (50.8%)	1014 (50.6%)	949 (49.4%)	936 (48.7%)	908 (47.3%)	776 (38.6%)
Female	7000 (52.2%)	951 (51.8%)	932 (49.2%)	925 (49.4%)	995 (50.6%)	1003 (51.3%)	1034 (52.7%)	1160 (61.4%)
Race								
White People	5870 (73.1%)	854 (73.1%)	936 (76.0%)	896 (75.4%)	897 (75.8%)	820 (72.1%)	826 (72.6%)	641 (65.3%)
Black People	2658 (9.1%)	412 (10.0%)	334 (7.6%)	323 (7.3%)	314 (7.2%)	332 (7.8%)	383 (8.9%)	560 (15.5%)
Hispanics	3720 (11.3%)	516 (10.8%)	501 (9.9%)	518 (10.9%)	522 (10.5%)	574 (12.8%)	530 (11.6%)	559 (13.2%)
Others	1335 (6.5%)	158 (6.1%)	172 (6.4%)	202 (6.4%)	211 (6.5%)	213 (7.3%)	203 (7.0%)	176 (6.0%)
**Income-poverty ratio**	3.25 ± 0.05	3.17 ± 0.08	3.28 ± 0.07	3.27 ± 0.06	3.34 ± 0.06	3.24 ± 0.07	3.26 ± 0.06	3.20 ± 0.07
<1.3	3812 (17.6%)	573 (19.0%)	549 (17.0%)	533 (16.9%)	522 (15.5%)	555 (18.7%)	524 (17.9%)	556 (18.6%)
1.3 to < 3.5	4474 (32.7%)	650 (33.7%)	622 (32.1%)	635 (33.5%)	644 (33.0%)	639 (31.6%)	640 (31.7%)	644 (33.1%)
≥ 3.5	4184 (49.7%)	557 (47.3%)	590 (51.0%)	624 (49.5%)	630 (51.5%)	589 (49.7%)	612 (50.4%)	582 (48.4%)
**BMI** (kg/m^2^)	29.5 ± 0.1	29.6 ± 0.2	29.7 ± 0.2	29.7 ± 0.2	29.8 ± 0.2	29.3 ± 0.2	29.4 ± 0.2	29.2 ± 0.3
< 25	3201 (24.6%)	425 (23.5%)	416 (20.8%)	447 (23.0%)	441 (24.0%)	487 (26.7%)	457 (25.5%)	528 (29.8%)
≥ 25 to < 30	4653 (35.2%)	689 (36.9%)	683 (37.8%)	671 (34.7%)	674 (34.8%)	650 (34.9%)	651 (34.5%)	635 (32.8%)
≥ 30	5569 (40.1%)	799 (39.6%)	818 (41.5%)	791 (42.2%)	806 (41.2%)	784 (38.4%)	816 (39.9%)	755 (37.5%)
**eGFR** (ml/min/1.73 m^2^)	89.8 ± 0.3	86.1 ± 0.6	87.8 ± 0.5	89.4 ± 0.6	90.4 ± 0.5	90.5 ± 0.5	91.6 ± 0.5	92.9 ± 0.5
≥ 90	7250 (55.1%)	891 (48.3%)	934 (50.7%)	1017 (53.4%)	1085 (57.2%)	1092 (57.3%)	1101 (58.5%)	1130 (60.6%)
60-89.9	5271 (38.9%)	787 (40.6%)	803 (41.0%)	773 (41.1%)	738 (38.2%)	724 (37.4%)	742 (37.7%)	704 (36.1%)
30-59.9	1062 (6.0%)	262 (11.1%)	206 (8.3%)	149 (5.5%)	121 (4.6%)	123 (5.3%)	99 (3.8%)	102 (3.3%)
Smoking status								
Never	6988 (51.7%)	863 (44.5%)	927 (50.0%)	954 (50.7%)	996 (50.1%)	1022 (52.6%)	1097 (55.3%)	1129 (59.0%)
Former	3919 (29.8%)	609 (33.0%)	617 (30.9%)	577 (29.6%)	574 (30.9%)	549 (30.3%)	515 (28.3%)	478 (25.1%)
Current	2668 (18.5%)	463 (22.5%)	399 (19.1%)	407 (19.6%)	374 (19.0%)	367 (17.1%)	329 (16.4%)	329 (16.0%)
**Diabetes**	2530 (14.0%)	432 (17.0%)	341 (12.8%)	323 (14.1%)	349 (13.4%)	342 (12.9%)	347 (13.2%)	396 (14.8%)
**Hypertension**	6250 (41.4%)	992 (46.3%)	949 (42.4%)	842 (39.6%)	858 (40.7%)	847 (12.9%)	897 (42.1%)	865 (39.9%)
**Cardiovascular disease**	1403 (8.6%)	268 (11.2%)	219 (8.7%)	199 (8.6%)	201 (8.0%)	189 (8.8%)	165 (7.4%)	162 (7.2%)
**Previous stroke**	592 (3.2%)	101 (3.3%)	107 (5.1%)	89 (3.2%)	80 (2.8%)	72 (2.9%)	71 (2.7%)	72 (2.4%)
**HCV infection**	338 (2.1%)	38 (1.6%)	34 (1.2%)	35 (1.7%)	37 (1.8%)	47 (1.9%)	57 (2.8%)	90 (4.0%)

eGFR, estimated glomerular filtration rate; BMI, body mass index; HCV, hepatitis C virus.

**Table 2:** Factors associated with soluble α-klotho levels by weighted multiple linear regression analysis.

Parameter	Estimate (B)	Std. Error	95% CI lower bound	95% CI upper bound	*p* value
Intercept	709.3	38.7	632.2	786.3	<0.001
Age	-0.9	0.4	-1.6	-0.2	<0.05
Female (vs. male)	35.1	6.5	22.2	48.0	<0.001
Race/ethnicity					
White People	Reference				
Black People	85.6	12.4	60.9	110.2	<0.001
Hispanic	9.5	9.7	-9.7	28.7	>0.05
Other	-4.4	12.4	-29.1	20.4	>0.05
BMI	-1.6	0.6	-2.7	-0.5	<0.01
eGFR	1.7	0.2	1.3	2.1	<0.001
Smoking status					
Never	65.1	9.3	46.6	83.7	<0.001
Former	38.7	9.4	19.9	57.5	<0.001
Current smoker	Reference				
Diabetes	10.1	9.6	-9.0	29.3	>0.05
Hypertension	2.6	7.1	-11.6	16.7	>0.05
Cardiovascular disease	-0.3	10.1	-20.5	19.8	>0.05
Stroke	-16.7	12.7	-42.0	8.5	>0.05
HCV infection	171.4	39.9	91.9	250.9	<0.001

eGFR, estimated glomerular filtration rate; BMI, body mass index; HCV, hepatitis C virus.

**Figure 1:**
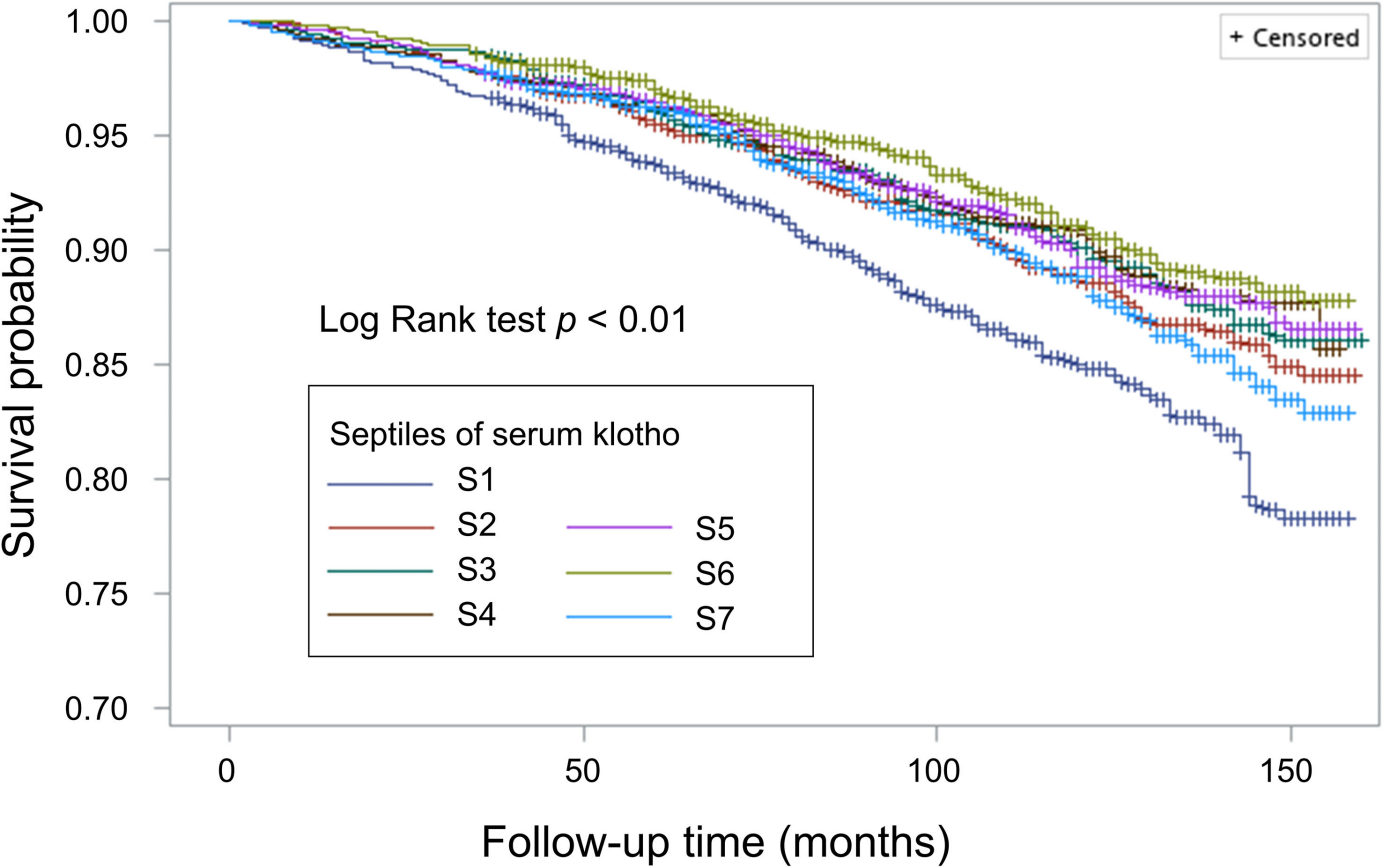
Survival curve for all-cause mortality by septile of soluble α-klotho.

During a median follow-up of 99.0 months (interquartile range: 68.0-129.0 months), a total of 1,490 participants died (11.8 per 10000 person-months). By Kaplan-Meier method, we observed that individuals in the lowest septile of soluble α-klotho level had higher risk of all-cause mortality (shown in **Figure 1**). Using weighted Cox regression analysis with adjustment for age and sex (model 1 in **Table 3**), our results showed that group 2 (HR: 0.72, 95% CI 0.58-0.93, p<0.05), group 3 (HR: 0.68, 95% CI 0.52-0.90, p<0.01), group 4 (HR: 0.69, 95% CI 0.54-0.88, p<0.01), group 5 (HR: 0.70, 95% CI 0.55-0.89, p<0.01), and group 6 (HR: 0.64, 95% CI 0.47-0.86, p<0.01) were associated with lower all-cause mortality risk compared with group 1; however, we did not observe a significant difference in mortality risk between group 1 and group 7 (**Table 3**). The association remained consistent after further adjustment for race/ethnicity, BMI, survey cycle, eGFR, diabetes, hypertension, CVD, stroke, HCV infection, smoking status, and family income-to-poverty ratio (model 2, model 3, and model 4 in **Table 3**).

**Table 3:** All-cause mortality risk by septile of soluble α-klotho levels .

Parameter		Crude model	Model 1	Model 2	Model 3	Model 4
	No. of events	HR (95% CI)	HR (95% CI)	HR (95% CI)	HR (95% CI)	HR (95% CI)
All-cause mortality						
S1 (n=1940)	297	Reference	Reference	Reference	Reference	Reference
S2 (n=1943)	226	0.71 (0.56-0.90) **	0.72 (0.57-0.93) *	0.75 (0.58-0.98) *	0.75 (0.57-0.97) *	0.74 (0.57-0.97) *
S3 (n=1939)	202	0.64 (0.47-0.86) **	0.68 (0.52-0.90) **	0.71 (0.53-0.96) *	0.72 (0.54-0.97) *	0.71 (0.53-0.96) *
S4 (n=1944)	193	0.62 (0.48-0.79) ***	0.69 (0.54-0.88) **	0.69 (0.54-0.89) **	0.69 (0.54-0.89) **	0.69 (0.53-0.89) **
S5 (n=1939)	198	0.62 (0.49-0.79) ***	0.70 (0.55-0.89) **	0.72 (0.56-0.92) **	0.76 (0.59-0.98) *	0.73 (0.57-0.94) *
S6 (n=1942)	157	0.54 (0.40-0.73) ***	0.64 (0.47-0.86) **	0.64 (0.48-0.86) **	0.66 (0.49-0.89) **	0.65 (0.48-0.90) **
S7 (n=1936)	217	0.73 (0.59-0.90) **	0.98 (0.79-1.21)	0.98 (0.79-1.22)	1.00 (0.81-1.23)	0.95 (0.76-1.18)

Model 1: adjusted for age and sex.

Model 2: adjusted for age, sex, race/ethnicity, body mass index (BMI), survey cycle, estimated glomerular filtration rate (eGFR), diabetes, hypertension.

Model 3: adjusted for age, sex, race/ethnicity, BMI, survey cycle, eGFR, diabetes, hypertension, cardiovascular disease (CVD), stroke, hepatitis C virus (HCV) infection, smoking status.

Model 4: adjusted for age, sex, race/ethnicity, BMI, survey cycle, eGFR, diabetes, hypertension, CVD, stroke, HCV infection, smoking status, family income-to-poverty ratio.

*p<0.05; **p<0.01; ***p<0.001.

**Figure 2:**
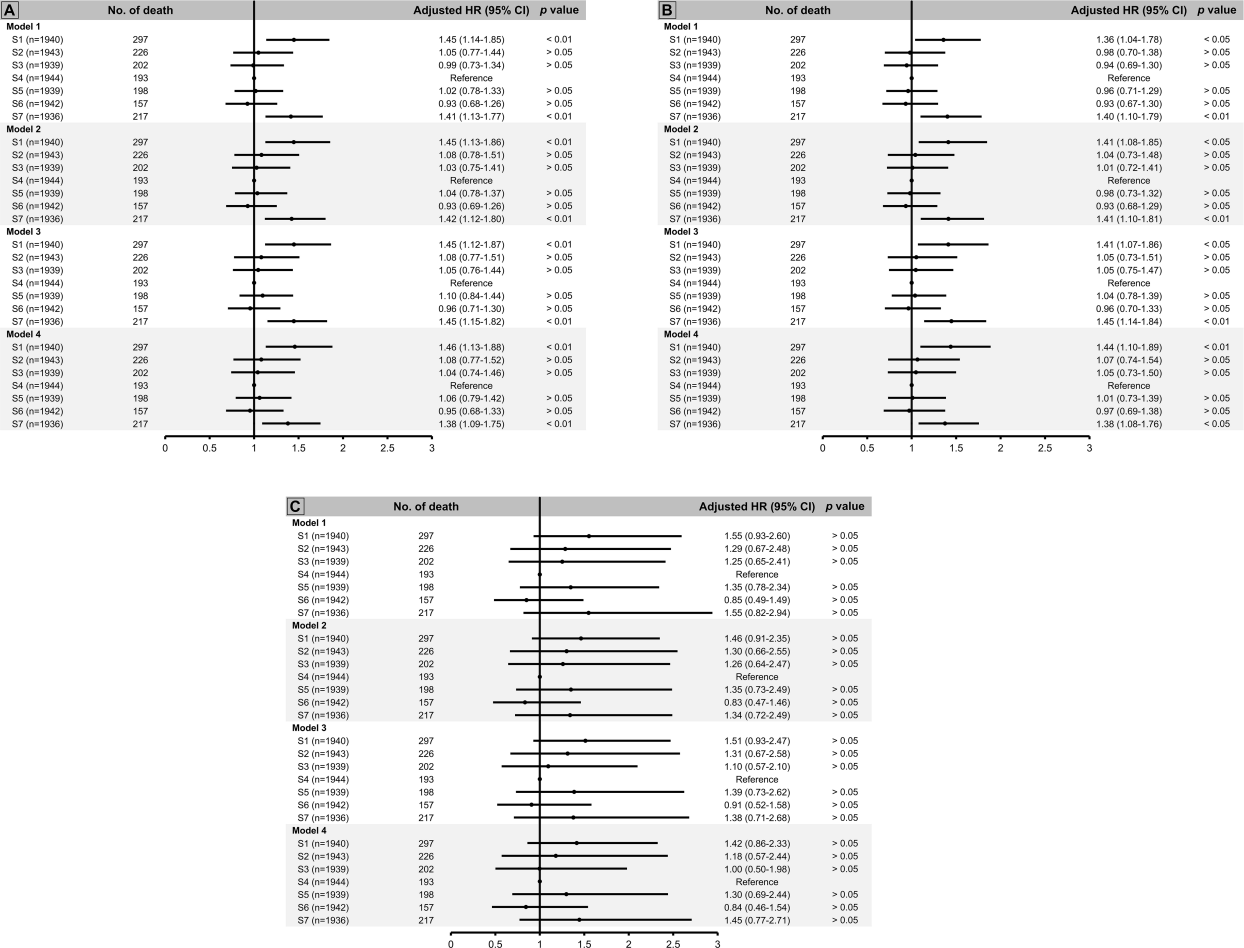
All-cause mortality risk by septile of soluble α-klotho using S4 as reference group among **(A)** total population, **(B)** individuals with estimated glomerular filtration rate (eGFR) ≥ 60 ml/min/1.73 m^2^, and **(C)** those with eGFR < 60 ml/min/1.73 m^2^. Model 1: adjusted for age and sex. Model 2: adjusted for age, sex, race/ethnicity, body mass index (BMI), survey cycle, estimated glomerular filtration rate (eGFR), diabetes, hypertension. Model 3: adjusted for age, sex, race/ethnicity, BMI, survey cycle, eGFR, diabetes, hypertension, cardiovascular disease (CVD), stroke, hepatitis C virus (HCV) infection, smoking status. Model 4: adjusted for age, sex, race/ethnicity, BMI, survey cycle, eGFR, diabetes, hypertension, CVD, stroke, HCV infection, smoking status, family income-to-poverty ratio.

Using group 4 as the reference group, we observed that both group 1 and group 7 were associated with 40% greater risk of death (shown in **Figure 2A**). In addition, when we stratified our analysis by kidney function, we observed a significant biphasic association between soluble α-klotho level and mortality risk in individuals with eGFR ≥ 60 mL/min/1.73 m^2^, but there was no significant association between α-klotho level and mortality risk in those with eGFR < 60 mL/min/1.73 m^2^ (shown in **Figures 2B, C**).

Using multinomial logistic regression analysis, we found that individuals in group 1 were more likely to be former or current smokers, Black people, and have lower BMI and lower eGFR compared with those in group 4 (**Table 4**). On the other hand, individuals in group 7 were more likely to be female, Black people or Hispanics, have lower BMI and higher eGFR, have HCV infection, and were less likely to be former or current smokers compared with those in group 4 (**Table 4**).

**Table 4:** Characteristic differences between groups of soluble α-klotho levels by multinomial logistic regression using the 4th septile (S4) as reference group.

	S1	S2	S3	S5	S6	S7
	OR (95% CI)	OR (95% CI)	OR (95% CI)	OR (95% CI)	OR (95% CI)	OR (95% CI)
**Age** (10 years)	1.02 (0.92-1.14)	0.98 (0.89-1.09)	0.99 (0.90-1.08)	0.98 (0.90-1.08)	0.96 (0.86-1.07)	0.93 (0.83-1.03)
**Female (vs. male)**	1.06 (0.89-1.26)	0.92 (0.76-1.10)	0.94 (0.78-1.14)	1.02 (0.84-1.23)	1.06 (0.88-1.28)	1.53 (1.25-1.87) ***
Race						
Black (vs. White)	1.28 (1.02-1.62) *	0.93 (0.74-1.16)	1.02 (0.81-1.27)	1.15 (0.92-1.44)	1.31 (1.06-1.62) *	2.51 (1.97-3.21) ***
Hispanics (vs. White)	1.17 (0.93-1.46)	0.98 (0.79-1.21)	1.07 (0.87-1.33)	1.23 (0.99-1.51)	1.05 (0.87-1.27)	1.24 (1.00-1.53) *
Others (vs. White)	0.96 (0.68-1.35)	1.09 (0.77-1.52)	1.03 (0.75-1.43)	1.17 (0.90-1.52)	1.03 (0.77-1.38)	0.96 (0.70-1.32)
**Income-poverty ratio**	0.98 (0.92-1.05)	0.98 (0.93-1.04)	0.98 (0.94-1.03)	0.97 (0.92-1.03)	0.97 (0.92-1.03)	0.99 (0.94-1.05)
**BMI** (kg/m^2^)	0.99 (0.97-1.00) *	1.00 (0.99-1.01)	1.00 (0.98-1.01)	0.99 (0.97-1.01)	0.99 (0.97-1.00)	0.98 (0.96-1.00) *
**eGFR** (10 ml/min/1.73 m^2^)	0.88 (0.84-0.93) ***	0.92 (0.86-0.98) *	0.96 (0.91-1.02)	1.00 (0.95-1.06)	1.04 (0.98-1.10)	1.09 (1.03-1.16) **
Smoking status						
Former (vs. never)	1.23 (1.04-1.45) *	1.01 (0.83-1.22)	0.97 (0.80-1.18)	0.98 (0.78-1.24)	0.90 (0.73-1.10)	0.78 (0.64-0.93) **
Current (vs. never)	1.41 (1.07-1.86) *	1.00 (0.76-1.31)	1.02 (0.81-1.29)	0.80 (0.63-1.01)	0.72 (0.56-0.94) *	0.61 (0.47-0.80) ***
**Diabetes**	1.23 (0.98-1.54)	0.87 (0.67-1.12)	1.01 (0.77-1.34)	0.99 (0.75-1.31)	1.03 (0.78-1.35)	1.25 (0.94-1.67)
**Hypertension**	1.12 (0.93-1.34)	1.00 (0.81-1.23)	0.93 (0.77-1.12)	0.94 (0.78-1.14)	1.15 (0.97-1.36)	0.99 (0.79-1.24)
**Cardiovascular disease**	1.15 (0.86-1.55)	0.91 (0.67-1.23)	1.00 (0.74-1.34)	1.15 (0.82-1.61)	0.94 (0.67-1.32)	1.20 (0.88-1.64)
**Previous stroke**	0.82 (0.58-1.16)	1.69 (1.07-2.67) *	1.13 (0.80-1.60)	0.98 (0.69-1.39)	1.06 (0.68-1.66)	0.94 (0.63-1.41)
**HCV infection**	0.86 (0.40-1.82)	0.54 (0.27-1.07)	1.02 (0.55-1.88)	1.12 (0.60-2.12)	1.39 (0.78-2.49)	2.64 (1.42-4.93) **

eGFR, estimated glomerular filtration rate; BMI, body mass index; HCV, hepatitis C virus. *p<0.05; **p<0.01; ***p<0.001.

## Discussion

In this population-based cohort study, our results showed that male sex, current smoking, older age, higher BMI, and lower eGFR were associated with lower soluble α-klotho levels, while HCV infection significantly correlated to higher soluble α-klotho levels. In addition, we observed a non-linear relationship between soluble α-klotho levels and mortality risk, with individuals in the lowest and highest septile at increased risk of all-cause mortality compared to those in the middle septile. When stratified by kidney function, we showed that the U-shaped association between soluble α-klotho levels and mortality risk was significant in those with eGFR ≥ 60 mL/min/1.73 m^2^, but not in those with eGFR < 60 mL/min/1.73 m^2^.

Previous research on the association between α-klotho levels and mortality risk had yielded inconsistent results. Among patients with pre-dialysis CKD or end-stage kidney disease (ESKD) undergoing maintenance hemodialysis, several studies suggested that a low soluble klotho level correlated with an increased risk of all-cause mortality (23–27), while others reported no significant differences in mortality risk between individuals with low and high levels of klotho (28–31). Although a meta-analysis suggested that a lower serum klotho level significantly correlated with an increased risk of all-cause mortality in CKD patients (20), substantial heterogeneity and relatively small sample sizes in each study had precluded a robust conclusion. Our study showed that serum α-klotho level and the risk of death were not strictly inversely correlated, which might explain the inconsistent findings in previous studies. In addition, klotho is composed of three isoform categories (membrane klotho, secreted klotho, and soluble klotho), and the detection of klotho by immunostaining can be affected by cross-reactivity and may not always be specific due to inter-assay variability (32). The non-specificity and cross-reactivity may have led to measurement bias and the conflicting results in previous studies. Although the detection of soluble α-klotho in NHANES was limited to a single commercial ELISA kit, whether measurement bias has affected our findings remains to be clarified.

The U-shaped dose-response relationship in biology was commonly observed in clinical and public health studies, and it had been postulated that this biphasic response might reflect a combined agonist-antagonist effect of the biological substance on its receptor system or the result of compensation for a disruption of homeostasis (33). A cohort study using soluble α-klotho as a continuous variable similarly showed a U-shaped relationship between klotho level and mortality risk among diabetic population (34), which was consistent with our findings. In addition, in patients without or with early CKD, a U-shaped association between klotho level and risk of fracture had also been observed (35, 36). Given the complex roles of klotho in regulation of mineral and vitamin D metabolism (37), our findings may suggest that individuals at both extremes of soluble α-klotho may have disrupted physiological homeostasis and thus be at higher risk of mortality.

Our study showed that HCV infection was significantly associated with higher soluble α-klotho levels, whereas other comorbidities including diabetes, hypertension, CVD, and previous stroke were not. Previous studies had suggested that an increased level of klotho could represent a compensatory response to physiologic stress and inflammation (38, 39). In alcoholic cirrhotic patients, studies showed higher-than-normal levels of soluble α-klotho in association with liver function derangement, possibly reflecting an attempt to regulate increased inflammation (40). In addition, in alcoholic individuals, those with higher klotho levels were found to have a greater mortality risk compared to those with lower levels, and this correlation was even more pronounced in cirrhotic patients (39). Whether our observation that HCV infection was associated with higher soluble α-klotho levels involved similar mechanisms remains to be elucidated.

Our study using a nationally representative population added to the evidence for the complex association between soluble α-klotho levels and long-term mortality risk. However, there are some limitations that should be considered when interpreting our findings. First, serum α-klotho was measured using stored surplus serum and therefore sample quality may have introduced measurement bias. Second, sociodemographic factors and comorbidity status were determined by participants’ self-reports, which may have influenced the accuracy of the measurements. Finally, despite our effort to adjust for several potential confounders, residual confounding may still exist and affect our results. Given the observational nature of our study, the causality between soluble α-klotho levels and mortality risk cannot be confirmed.

In summary, among middle-aged and older U.S. adults, our study showed a U-shaped association between soluble α-klotho level and all-cause mortality risk, especially for those with eGFR ≥ 60 mL/min/1.73 m^2^. While there is no established normal range for soluble α-klotho, our findings suggest that individuals with levels ≤ 575.8 or ≥ 1140.1 pg/ml are both at increased risk of death. The extremely high soluble α-klotho levels may represent a compensatory response to physiological stress and chronic inflammation and therefore correlate to a poor prognosis. Further research is warranted to see if our findings can be reproduced and to explore the underlying mechanisms. In addition, it is important to consider whether the increased mortality risk observed at the highest extreme of soluble α-klotho would affect the findings of studies exploring the therapeutic potential of klotho.

## Data availability statement

Publicly available datasets were analyzed in this study. This data can be found at: https://wwwn.cdc.gov/nchs/nhanes/Default.aspx.

## Ethics statement

The studies involving humans were approved by the research ethics review board of the NCHS. The studies were conducted in accordance with the local legislation and institutional requirements. The participants provided their written informed consent to participate in this study.

## Author contributions

Research concept and study design: M-YJ and M-HC. Data acquisition: M-YJ. Data analysis/interpretation: M-HC, H-WW, Y-TH, and M-YJ. Statistical analysis: M-YJ. Supervision: M-YJ. Each author contributed intellectual content during the manuscript drafting/revision process and accepts accountability for the overall work and ensuring that questions pertaining to the accuracy or integrity of any portion of the work are appropriately investigated and resolved. M-YJ had full access to all study data and take responsibility for data integrity and data analysis accuracy. All authors contributed to the article and approved the submitted version.
